# Unravelling the Features of Success of VIM-Producing ST111 and ST235 *Pseudomonas aeruginosa* in a Greek Hospital

**DOI:** 10.3390/microorganisms8121884

**Published:** 2020-11-28

**Authors:** Costas C. Papagiannitsis, Aggeliki Verra, Vasiliki Galani, Stelios Xitsas, Ibrahim Bitar, Jaroslav Hrabak, Efthymia Petinaki

**Affiliations:** 1 Department of Microbiology, University Hospital of Larissa, 41110 Larissa, Greece; jlkverra@yahoo.gr (A.V.); galanivasiliki@gmail.com (V.G.); stexit007@gmail.com (S.X.); petinaki@uth.gr (E.P.); 2 Biomedical Center, Faculty of Medicine, Charles University, 32300 Pilsen, Czech Republic; ibrahimbitar5@gmail.com (I.B.); jaroslav.hrabak@lfp.cuni.cz (J.H.)

**Keywords:** VIM, ST235, ST111, Illumina sequencing, class 1 integrons, CRISPR/Cas system

## Abstract

The objective of this study was to analyze the characteristics that contribute to the successful dissemination of VIM-producing *Pseudomonas aeruginosa* (*P. aeruginosa*), belonging to ST111 and ST235, in a Greek hospital. A total of 120 non-repetitive *P. aeruginosa*, which had meropenem minimal inhibitory concentrations (MICs) greater than 2 mg/L, were studied. VIM-encoding genes were amplified and sequenced within their integrons. Isolates were typed by multilocus sequence typing (MLST). Six VIM-producers, representative of different integron structures and sequence types (STs), were completely sequenced using Illumina platform. Sixty-one *P. aeruginosa* were confirmed to produce VIM-type carbapenemases. ST111 dominated (*n* = 34) among VIM-producers, while 15 VIM-producers belonged to ST235. The *bla*_VIM_-like genes were located in three integron types, including In59, In595 and In1760, which were integrated into *P. aeruginosa* chromosomes. Whole genome sequencing (WGS) data demonstrated that ST111 and ST235 MBL producers carried several resistance and virulence genes. Additionally, the presence of type I-C and type I-E clustered regularly interspaced short palindromic repeats (CRISPR)/Cas locus was observed in ST235 and ST395 isolates, respectively. In conclusion, our findings confirmed the clonal spread of ST111 *P. aeruginosa*, carrying the VIM-2-encoding integron In59, in the University Hospital of Larissa (UHL). In addition, they highlighted the important role of high-risk clones, ST111 and ST235, in the successful dissemination and establishment into hospital settings of clinically important pathogens carrying resistance determinants.

## 1. Introduction

*Pseudomonas aeruginosa* is an opportunistic pathogen causing a wide range of infections, including pulmonary infections, wound and sepsis. *P. aeruginosa* show a great tendency to form biofilms on medical devices [1]. Additionally, *P. aeruginosa* express virulence factors, like exotoxins, proteases and pigment. These factors help the bacterium to facilitate successful colonization and infection across a wide range of environments.

Furthermore, *P. aeruginosa* show a high prevalence of antimicrobial resistance. Approximately 13% of *P. aeruginosa*, causing infections, are multidrug-resistant (MDR) strains. Therefore, recently, the World Health Organization (WHO) has considered *P. aeruginosa* as a target of high priority for the development of new antibiotics [2].

The MDR phenotype is developed mainly through over-expression of the intrinsic AmpC-type cephalosporinase, inactivation or loss of the OprD porin and/or upregulation of efflux pumps. In addition, the acquisition of resistance genes, including those encoding β-lactamases, like extended-spectrum-β-lactamases (ESBLs) or metallo-β-lactamases (MBLs) [3], contributes to the development of the MDR phenotype. MBLs are zinc-dependent enzymes hydrolyzing all β-lactams, except monobactams. Their activity is not affected by the currently available β-lactamase inhibitors. Contrary to *bla*_NDM_ genes, *bla*_IMP_ and *bla*_VIM_ occur as gene cassettes in class 1 integrons [4]. These integrons often also carry genes conferring resistance to other antibiotics, like trimethoprim, aminoglycosides and chloramphenicol.

In Greece, the first outbreak of *P. aeruginosa* isolates harboring *bla*_VIM-1_ took place in AHEPA University Hospital of Thessaloniki during the period of 1996–1998 [5]. In 2001, carbapenem-resistant *P. aeruginosa* harboring the *bla*_VIM-2_ gene predominated among 15 Greek hospitals. In most of the cases, *bla*_VIM-2_ was found in the variable region of class 1 integron In56 or In59 [6]. Moreover, in 2001, *bla*_VIM-4_ was characterized from a clinical *P. aeruginosa* recovered in the University Hospital of Larissa (UHL) [7], central Greece. During 2001–2002, in UHL, the frequency of carbapenem-resistant *P. aeruginosa* reached 35%, and *P. aeruginosa* producing VIM-2 or VIM-4 MBLs predominated [8]. According to the multilocus sequence typing (MLST) Oxford scheme, ST111 and ST235 have been the prevalent types among VIM-producing *P. aeruginosa* in our region [9].

The last data published from our institution referred to MBL-producing *P. aeruginosa* collected from central Greece, in 2011 [9]. However, it is important to continuously monitor and study the molecular epidemiology of carbapenem-resistant *P. aeruginosa*, which constantly evolves in a global level, especially in endemic areas as these regions constitute the focus for such strains. Therefore, the molecular epidemiology of MBL-producing *P. aeruginosa* collected in 2018 from patients treated in UHL was investigated. In addition, we aimed to analyze the characteristics that contribute to the success of epidemic clones ST111 and ST235.

## 2. Materials and Methods 

### 2.1. Clinical Isolates, Identification and Susceptibility Testing

A collection of nonrepetitive 120 *P. aeruginosa*, isolated in 2018 from patients treated in UHL, was analyzed in this study. For each month, ten first isolates that had minimal inhibitory concentration (MIC) to meropenem greater than 2 mg/L according to 2019 EUCAST breakpoints were selected. Identification of clinical isolates was performed by the VITEK-2 system (bioMérieux, Marcy l’Étoile, France). Antimicrobial susceptibility of bacterial isolates against several antibiotics including piperacillin, piperacillin–tazobactam, cefotaxime, ceftazidime, cefepime, aztreonam, imipenem, meropenem, gentamicin, amikacin, tobramycin, chloramphenicol, tetracycline, trimethoprim-sulfamethoxazole, ciprofloxacin, and levofloxacin was also performed by the VITEK-2 system. The MICs of colistin were determined by the broth dilution method [10]. *P. aeruginosa* ATCC 27853 and *Escherichia coli* ATCC25922 were used as control strains [11]. Susceptibility data were interpreted according to the criteria (version 9.0) of the European Committee on Antimicrobial Susceptibility Testing (EUCAST) (www.eucast.org).

### 2.2. Confirmation of Carbapenemase Production

All isolates were subjected to phenotypic detection of MBLs using DDST with EDTA [12]. Genes encoding KPC, NDM, VIM, IMP and OXA-48-like carbapenemases were detected by PCR amplification [13].

### 2.3. Multilocus Sequence Typing

All *P. aeruginosa* isolates were analyzed by multilocus sequence typing (MLST) [14]. The database at https://pubmlst.org/paeruginosa was employed for determining STs.

### 2.4. Integron Analysis

Variable regions of class 1 integrons with *bla*_VIM_-like genes were amplified in two parts, from the 5′ conserved segment (5′CS) to *bla*_VIM_ cassette, and from *bla*_VIM_ cassette to the 3′ conserved segment (3′CS) [13]. VIM-encoding integrons were sequenced using an ABI 3500 sequencer (Applied Biosystems, Foster City, CA, USA). Integron sequences were analyzed using the Integrall integron database (http://integrall.bio.ua.pt) [15].

### 2.5. Plasmid Analysis

Six VIM-producing *P. aeruginosa* (VPP) were selected as representatives of all different STs and integron types in order to define the genetic units harboring the *bla*_VIM_-like genes. The plasmid contents of the selected isolates were analyzed by pulsed-field gel electrophoresis (PFGE) of total DNA digested with S1 nuclease (Promega, Madison, WI, USA) [16], followed by *bla*_VIM_ hybridization [13].

### 2.6. Whole Genome Sequencing

The 6 VPP isolates, used in “plasmid analysis’ experiments” were further analyzed with whole genome sequencing (WGS). For comparison purposes, nine non-carbapenemase-producing *P. aeruginosa* were also included. The genomic DNAs of *P. aeruginosa* were extracted using the DNA-Sorb-B kit (Sacace Biotechnologies S.r.l., Como, Italy). Multiplexed DNA libraries were prepared, using the Nextera XT Library Preparation Kit (Illumina Inc., San Diego, CA, USA), and 300-bp paired-end sequencing was performed on Illumina MiSeq platform (Illumina Inc., San Diego, CA, USA) using the MiSeq v3 600-cycle Reagent Kit (Illumina Inc., San Diego, CA, USA). Initial paired-end reads were quality trimmed using Trimmomatic tool v0.32. Then, reads were assembled via de Bruijn graph-based de novo assembler SPAdes v3.14.0. The sequence gaps were confirmed by PCR-based strategy and Sanger sequencing of the amplicons. For sequence analysis and annotation, the BLAST algorithm (www.ncbi.nlm.nih.gov/BLAST), the open reading frame (ORF) finder tool (www.bioinformatics.org/sms/) and the ISFinder database (www-is.biotoul.fr/) were employed. Comparative genome alignment was conducted using Mauve v.2.3.1. (http://darlinglab.org/mauve/mauve.html).

WGS data were analyzed using the ResFinder 3.2 tool (https://cge.cbs.dtu.dk/services/ResFinder/), with an identity threshold >90% [17], and with the Virulence Factors Database (VFDB; http://www.mgc.ac.cn/VFs/main.htm) [18]. Finally, the CRISPRCasFinder program (https://crisprcas.i2bc.paris-saclay.fr/CrisprCasFinder/Index) was utilized for the easy detection of clustered regularly interspaced short palindromic repeats (CRISPR) and Cas-associated genes [19].

### 2.7. Nucleotide Sequence Accession Numbers

Nucleotide sequences representing the genetic context of VIM-encoding integrons In595 and In1760 were submitted to the GenBank under accession numbers MT428323 and MT437279, respectively.

## 3. Results

Twenty-four *P. aeruginosa* isolates had MIC to meropenem of 4–8 mg/L and were categorized as susceptible at increased exposure, whereas 96 isolates had MIC >8 mg/L and were categorized as resistant according to the 2019 EUCAST definition. The majority of *P. aeruginosa* isolates were resistant to cefepime (*n* = 87, 72.5%), ceftazidime (*n* = 93, 77.5%), imipenem (*n* = 96, 80%), piperacillin–tazobactam (*n* = 109, 90.8%) and piperacillin (*n* = 114, 95.0%). Only 62 (51.7%) isolates were resistant to aztreonam. One-hundred eight (90.0%) of *P. aeruginosa* isolates also exhibited resistance to levofloxacin, 104 (86.7%) were resistant to ciprofloxacin, 92 (76.7%) to tobramycin, 78 (65.0%) to amikacin, and 70 (58.3%) to gentamicin.

In 70 *P. aeruginosa* isolates, the EDTA–meropenem test was positive, indicating MBL production. However, 61 out of 70 the isolates, being positive in the EDTA-meropenem test, were positive for *bla*_VIM_ genes. Other types of carbapenemase-encoding genes were not detected.

**Figure 1:**
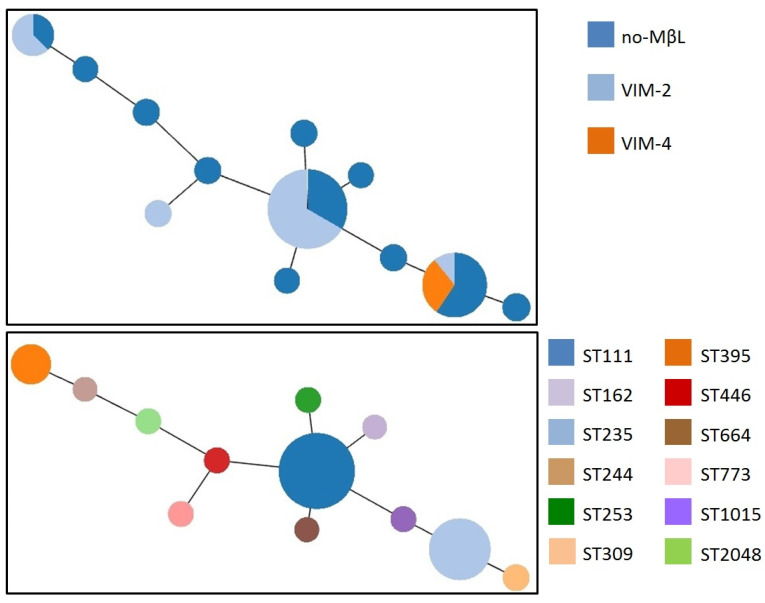
Minimal spanning tree of 120 *Pseudomonas aeruginosa* isolates, recovered from University Hospital of Larissa (UHL) during 2018, showing sequence types (STs) and carbapenemase content. Each circle corresponds to an ST. The area of each circle is proportional to the number of isolates.

The population structure of *P. aeruginosa* isolates studied by MLST showed that the VPP isolates included four sequence types (STs) (Figure 1). ST111 was the most predominant comprising 34 VIM-producing isolates (Table 1). Twenty five VIM producers were distributed in STs 235 (*n* = 15) and 395 (*n* = 10). STs 111, 235 and 395 have been assumed as “high-risk clones” [20]. The remaining VIM-producing *P. aeruginosa* isolates belonged to ST773 (*n* = 2). However, higher genetic diversity was observed in the group of non-carbapenemase-producing isolates (Table 1). This group of isolates (*n* = 59) comprised 11 clones, with STs 235 (*n* = 14) and 111 (*n* = 11) accounting for 25 isolates.

Characterization of the regions carrying the carbapenemase-encoding genes revealed that *bla*_VIM_-like genes were located in three main types of class 1 integrons (Table 1). The most common integron was In59 identified in 40 *P. aeruginosa*, including 34 ST111, 4 ST235 and 2 ST773 isolates. In59 possessed *aacA29a*, *bla*_VIM-2_ and *aacA29b* gene cassettes [21]. However, the 11 remaining ST235 VIM producers carried the class 1 integron In595 [22], including an array of *bla*_VIM-4_, *arr-7*, *aacA4*, *bla*_PSE-1_ cassettes. In595 has been previously described in two MBL-producing isolates imported from Greece and Cyprus in Scandinavia. One of those *P. aeruginosa* isolates was also ST235. Finally, among ST395 VIM producers, the class 1 integron In1760, whose variable region comprised the *gcu205*, *bla*_OXA-10_, *aacA4*, *bla*_VIM-2_, and *smr-2* cassettes, was identified. The *gcu205* cassette encoded a hypothetical protein of unknown function. The *bla*_OXA-10_ and *bla*_VIM-2_ genes mediated resistance to β-lactams, while the *aacA4* cassette conferred to aminoglycosides. The last cassette, *smr-2*, encoded a small multi-drug resistance protein mediating efflux of lipophilic cations. The majority (*n* = 57) of VIM-producing isolates were resistant to meropenem. However, four VIM-2 producers (three ST111 and one ST395 isolates) exhibited meropenem MIC 4–8 mg/L and were categorized as susceptible at increased exposure.

S1 profiling did not observer any DNA bands that could be assigned to plasmids, and the *bla*_VIM_-like probe hybridized with only the largest DNA bands, indicating the chromosomal location of the VIM-encoding integrons. Thus, the genomes of these isolates (Table 2) were sequenced by the Illumina platform. Sequence analysis showed that, in all cases, VIM-encoding integrons were inserted into *P. aeruginosa* chromosomes.

The In59-like integron was located in a Tn*5060*-like transposon integrated into the chromosome of ST111 *P. aeruginosa* isolates. However, insertion of the Tn*3*-like transposon Tn*4661* [23] probably deleted the Tn*5060 mer* module. Tn*4661* was found next to a partially deleted ICE1-like integrative conjugative element (ΔICE1), which was inserted into the tRNALys gene (PA4541.1 in GenBank accession no. AE004091). A similar structure has been observed in VIM-producing *P. aeruginosa* isolates belonging to ST111 isolated in Czech hospitals [13].

**Figure 2:**
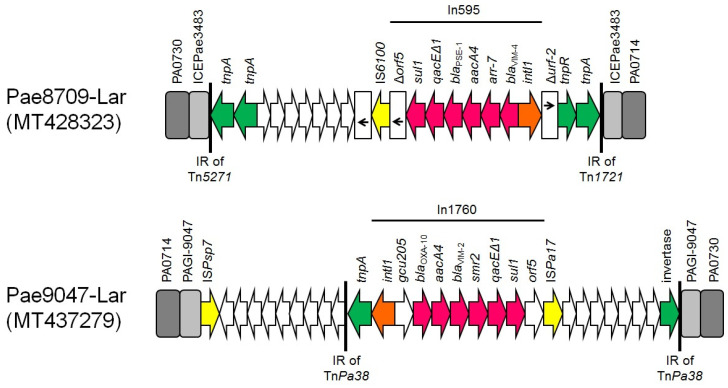
Linear maps of the genetic context of VIM-encoding class 1 integrons In595 and In1760 integrated into the *P. aeruginosa* chromosome. Arrows show the direction of transcription of open reading frames (ORFs), while truncated ORFs appear as rectangles (arrows within rectangles indicate the direction of transcription). Resistance genes, insertion sequences (IS), and transposases are shown in pink, yellow, and green, respectively. *intI1* genes are shaded orange. Sequences associated with genomic islands (GIs) are shaded light gray; dark gray rectangles indicate the *P. aeruginosa* chromosome. The remaining genes are shown in white.

In595 integron was in integrative conjugative element ICEPae3483, identified in the *P. aeruginosa* chromosome (Figure 2). ICEPae3483 was previously found in *P. aeruginosa* HSV3483 (GenBank accession no. MF168944) from Portugal. The In595 was embedded in a Tn*1721*-like transposon. The IRi of In595 was found in *tnpM* of the Tn*1721*-like, while the 3′CS of In595 was disrupted after the start codon of orf5 by an IS*6100* element.

In ST395 VIM-2 producers, In1760 was associated with a composite transposon, bounded by two copies of the IS*Psp7* insertion sequence in opposite orientation. The *bla*_VIM-2_-carrying transposon was inserted in a novel genomic island (GI) (Figure 2), designated PAGI-8709. PAGI-8709 included open reading frames (ORFs) encoding hypothetical proteins of unknown function and proteins of various functions (e.g., regulation of gene expression, DNA recombination and metabolic activities). GIs closely related to PAGI-8709 have not been found previously in *P. aeruginosa*. PAGI-8709 was integrated in *P. aeruginosa* chromosome into gene encoding for tRNALys (PA0729 in GenBank accession no. AE004091). Τhe 5′CS of the integron was intact, with the IRi of the integron being next to a 9685-bp segment (nucleotide 163493 to 166551 in GenBank accession no. MT437279), presenting 100% identity with α Tn*3*-like sequence from *Pseudomonas* sp. strain AG1 (GenBank accession no. CP045739), whereas an IS*Pa17* element was inserted into the 3′CS of the integron, downstream of *orf5*.

Additional genes conferring resistance to aminoglycosides, β-lactams and chloramphenicol were identified in the majority of the sequenced isolates (Table 2), as indicated by analysis of WGS data by the ResFinder 3.2 tool. Additionally, WGS data confirmed the absence of known carbapenemase-encoding genes in nine *P. aeruginosa* isolates being positive in the EDTA–meropenem test, but negative in PCR screening.

Examination of quinolone resistance-determining regions of *gyrA*, *gyrB*, *parC*, and *parE* and of the *mexR*, *nfxB*, and *mexT* genes, which regulate the MexAB-OprM, MexCD-OprJ, and MexEF-OprN multidrug efflux systems [24], revealed the presence of several point mutations predicted to result in several amino acid substitutions (Table 3). The majority of these amino acid substitutions have been identified previously from both ciprofloxacin-susceptible and ciprofloxacin-resistant *P. aeruginosa* isolates. However, in GyrA, the T83I amino acid substitution that previously has been associated with increased quinolone resistance [25] was found in all ciprofloxacin-resistant isolates. Furthermore, in the *oprD* gene, point mutations predicted to result in early termination of translation were identified in six *P. aeruginosa* isolates (Table 3). This finding is in agreement with increased resistance to carbapenems [26], even in non-carbapenemase-producing isolates.

Analysis of WGS data by VFDB showed that all sequenced isolates carried several virulence genes (Table 2) that can be involved in colonization and cause bloodstream invasion, extensive tissue damage and dissemination [27]. These included secreted toxins, like exoenzyme S, exoenzyme T, exoenzyme U and exoenzyme Y, subverting host cell defense and signaling systems [28]. Our results confirmed that the clone ST111 correlated with the copresence of *exoS*, *exoT*, and *exoY* genes, whose expression correlates with a higher risk of mortality [29]. On the other hand, clone ST235 correlated with the copresence of *exoT*, *exoU*, and *exoY*. In particular, ExoU is associated with lung damage and acute cytotoxicity [30]. Additionally, sequenced isolates carried genes encoding phospholipases (PlcH and PlcN), elastase B, alkaline protease A, exotoxin A and phenazines (Table 2), which have been previously associated with increased virulence of *P. aeruginosa* isolates [31,32].

Finally, the presence of the CRISPR/Cas system in *P. aeruginosa* isolates was examined. CRISPR/Cas systems provide bacteria with an adaptive immunity system against invading genetic elements [33], like bacteriophages and plasmids. A CRISPR/Cas system was found in seven of the sequenced *P. aeruginosa*. Isolates belonging to STs 235 and 395 exhibited type I-C and type I-E CRISPR/cas locus, respectively, while a type I-F CRISPR/Cas locus was found in ST244 and ST253 isolates (Table 2).

Both ST395 isolates exhibited identical CRISPR/Cas systems, further confirming their clonal relationship. The type I-E CRISPR/Cas locus consisted of eight genes consecutively encoding the Cas2, Cas1, Cas6, Cas5, Cas7, Cse2, Cse1 and Cas3 proteins. The locus enclosed two arrays of 12 (CRISPR1) and 8 spacers (CRISPR2). The spacers, presenting variable sequences, had a common length of 32 bp. Each spacer was flanked by two direct repeats (DRs), being 29 bp long. The DRs in CRISPR1 had the 5′-CGGTTCATCCCCACGCATGTGGGGAACAC-3′ consensus sequence, whereas the DRs in CRISPR2 had the 5′-CGGTTCATCCCCACACCCGTGGGGAACAC-3′ consensus sequence.

The type I-C CRISPR/Cas locus consisted of seven genes consecutively encoding the Cas3, Cas5, Cas8, Cas7, Cas4, Cas1, and Cas2 proteins. The locus was followed by an array of 20 spacers, whose variable sequences were 33–36 bp long. DRs flanking the spacers had the 5′-GTCGCGCCCCGCACGGGCGCGTGGATTGAAAC-3′ consensus sequence and a length of 32 bp. All ST235 isolates producing VIM-4 carbapenemase exhibited identical CRISPR/Cas systems. However, the type I-C CRISPR/Cas locus of the ST235 non-carbapenemase-producing isolate, Pae90-Lar, differed by an internal deletion erasing a spacer. This finding may indicate diverse evolution dynamics into those isolates. Type I-C locus was recently identified in ST235 isolates during a study analyzing the phylogenetic distribution of CRISPR/Cas systems in antibiotic-resistant *P. aeruginosa* [34].

In the ST244 isolate, Pae91-Lar, the type I-F CRISPR/Cas locus included six genes expressing the Cas1 endonuclease, Cas3/Cas2 helicase/RNAse, three Csy proteins (Csy1, Csy2, Csy3) and Cas6 endoribonuclease. The type I-F locus enclosed two arrays of 5 (CRISPR1) and 39 (CRISPR2) spacers. The spacers, presenting variable sequences, had a common length of 32 bp. Each spacer was flanked by two DRs 28 bp in length. The consensus sequence of the DRs found in CRIPSR1 was 5′-TTTTCTTAGCTGCCTATACGGCAGTGAAC-3′, whereas in CRISPR2, it was 5′-GTTCACTGCCGTGTAGGCAGCTAAGAAA-3′. Although a type I-F CRISPR/Cas locus was found in the ST253 isolate, Pae462-Lar, the locus exhibited 96.99% similarity to that identified in Pae91-Lar. Unlike Pae91-Lar, the locus included two arrays of 13 and 18 spacers. Type I-F locus was found previously in 38.5% of clinical *P. aeruginosa* isolates recovered from two Brazilian hospitals [35].

A BLASTn search with the identified spacers matched *P. aeruginosa* chromosomal sequences as well as different mobile elements, including bacteriophages and plasmids.

## 4. Discussion

In the current study, we analyzed the molecular characteristics of *P. aeruginosa* isolates belonging to the I and R susceptibility category according to meropenem MICs, recovered during surveillance program in UHL in 2018. A total of 50.8% of the isolates was confirmed to produce VIM-type carbapenemases. The majority (80.3%) of carbapenemase-producing isolates were assigned to “high-risk clones”, ST111 and ST235, while higher genetic diversity was observed in the group of non-carbapenemase-producing isolates, which were assigned to 11 different clones. Three main types of class 1 integrons carrying *bla*_VIM_-like genes were found. The VIM-2-encoding integron In59, which was firstly described in France in 1998 [21], was the most common integron type identified in *P. aeruginosa* of STs 111, 235 and 773. An In59-like integron was also the predominant type among ST111 *P. aeruginosa* isolates collected from UK hospital laboratories [36]. Additionally, the VIM-4-encoding integron In595 was identified in 11 ST235 *P. aeruginosa*, while the integron In1760, expressing VIM-2 MBL, was found among ST395 isolates.

In *P. aeruginosa* isolates analyzed with Illumina sequencing, WGS data confirmed the chromosomal location of VIM-encoding integrons. This finding is in agreement with the clonal spread of VIM-producing pathogens in our setting. Of note, a novel genomic island (designated as PAGI-8709) associated with the *bla*_VIM-2_-carrying integron In1760 was characterized from ST395 isolates. Additionally, WGS data confirmed the absence of carbapenemase-encoding genes in nine carbapenem-resistant *P. aeruginosa* that were positive in the EDTA–meropenem test. However, other traits corresponding to mutations in *mexR*, *nfxB*, and *mexT* genes, which regulate the MexAB-OprM, MexCD-OprJ, and MexEF-OprN multidrug efflux systems, and *oprD* were found in non-carbapenemase-producing isolates.

Furthermore, analysis of WGS data demonstrated that “high-risk clones”, ST111 and ST235, contributing to the spread of VIM-type carbapenemases in UHL exhibited a vast variety in their armamentariums, including resistance genes, virulence genes and/or a CRISPR/Cas system. Those features could be involved in the establishment of ST111 and ST235 isolates into hospital environments. The presence of a CRISCPR/Cas system was identified in carbapenemase-producing isolates, belonging to STs 235 and 395. Interestingly, both ST395 isolates exhibited identical type I-E CRISPR/Cas systems, further indicating their clonal relationship. A similar observation was also made for all ST235 VIM-4-producing isolates, which carried type I-C CRISPR/Cas systems. These findings restated that CRISPR/Cas systems can be used as tools for typing of bacteria. Bacteria harboring an active CRISPR/Cas system present a selective advantage against the income of harmful DNA [37]. However, this inhibition may have a negative impact on cases where horizontal gene transfer would benefit bacteria by making them more resistant. Recently, an additional function has been attributed to the CRISPR/Cas system in the regulation of genes associated with virulence [38].

## 5. Conclusions

In conclusion, this is the first study analyzing the presence of CRISPR/Cas systems in carbapenemase-producing *P. aeruginosa* isolates of Greek origin. In line with previous studies [34,35], our data confirmed the presence of type I-C, I-E and I-F systems in *P. aeruginosa* of clinical origin, and their strong association with specific lineages. Isolates, which belonged to the same clone, exhibited identical CRISPR/Cas systems underlined the fact that CRISPR arrays is a simple, but precise, genotyping tool that can be used to track pathogenic bacterium. Additionally, our findings that are consistent with the results of previous studies [13,20,39] highlighted the important role of high-risk clones, namely, STs 111 and 235, in the successful dissemination of clinically important resistance determinants. Isolates, belonging to STs 111 and 235 harbored a vast variety in their armamentariums, including resistance genes, virulence genes and/or a CRISPR/Cas system, which could be involved in their successful dissemination. Therefore, recognition of carbapenemase-producing *P. aeruginosa* hyper-epidemic clones by molecular tools, and more specifically of WGS [40], represents an important step towards tracing transmission routes, developing targeted control and prevention strategies, and monitoring their effectiveness.

## Figures and Tables

**Table 1:** Population structure of 120 *P. aeruginosa* isolates, recovered from UHL during 2018.

ST	MBL	No. of Isolates	Integrons with *bla*_VIM_ Genes
ST111	VIM-2	34	In59
	-	17	-
ST162	-	1	-
ST235	VIM-2	4	In59
	VIM-4	11	In595
	-	22	-
ST244	-	1	-
ST253	-	2	-
ST309	-	3	-
ST395	VIM-2	10	In1760
	-	6	-
ST446	-	2	-
ST664	-	1	-
ST773	VIM-2	2	In59
ST1015	-	2	-
ST2048	-	2	-

**Table 2:** Characteristics of 18 *P. aeruginosa* characterized by Illumina sequencing.

Isolate	ST	MBL Content	Integrons with *bla*_VIM_ Genes	Additional Resistance Genes	Virulence Genes	CRISPR/Cas System ^a^
Pae8397-Lar	111	VIM-2	In59	*bla*_PAO_*, bla*_OXA-395_, *aph*(3’)*-IIb*, *catB7*	*algD, aprA, lasB, plcH*, *plcN*, *exoS*, *exoT*, *exoY*, *phzM*, *phzS*, *phzH, pilA*, *pilB*, *toxA*	-
Pae9981-Lar	111	VIM-2	In59	*bla*_PAO_*, bla*_OXA-395_, *aph*(3’)*-IIb*, *catB7*	*algD, aprA, lasB, plcH*, *plcN*, *exoS*, *exoT*, *exoY*, *phzM*, *phzS*, *phzH, pilA*, *pilB*, *toxA*	-
Pae8709-Lar	235	VIM-4	In595	*bla*_PAO_*, bla*_OXA-35_, *bla*_OXA-488_, *aph*(3’)*-IIb*, *catB7*	*algD, aprA, lasB, plcH*, *plcN*, *exoT*, *exoU*, *exoY*, *phzM*, *phzS*, *phzH*, *pilB*, *toxA*	Type I-C
Pae9938-Lar	235	VIM-4	In595	*bla*_PAO_*, bla*_OXA-35_, *bla*_OXA-488_, *aph*(3’)*-IIb*, *catB7*	*algD, aprA, lasB, plcH*, *plcN*, *exoT*, *exoU*, *exoY*, *phzM*, *phzS*, *phzH*, *pilB*, *toxA*	Type I-C
Pae8414-Lar	395	VIM-2	In1760	*bla*_PAO_*, bla*_OXA-488_, *aadA6*, *aph*(3’)*-IIb*, aph(3’)*-Via*, *catB7*	*algD, aprA, lasB, plcH*, *plcN*, *exoS*, *exoT*, *exoY*, *phzM*, *phzS*, *phzH*, *pilB*, *toxA*	Type I-E
Pae9047-Lar	395	VIM-2	In1760	*bla*_PAO_*, bla*_OXA-488_, *aadA6*, *aph*(3’*)-IIb*, *aph*(3’)*-Via*, *catB7*	*algD, aprA, lasB, plcH*, *plcN*, *exoS*, *exoT*, *exoY*, *phzM*, *phzS*, *phzH, pilA*, *pilB*, *toxA*	Type I-E
Pae112-Lar	162	No	-	*bla*_PAO_*, bla*_OXA-494_, *aacA29*, *aph*(3’)*-IIb*, *catB7*, *sul1*	*algD, aprA, lasB, plcH*, *plcN*, *exoT*, *exoU*, *exoY*, *phzM*, *phzS*, *phzH*, *pilB*, *toxA*	-
Pae69-Lar	235	No	-	*bla*_PAO_*, bla*_OXA-2_, *bla*_OXA-488_, *bla*_PER-1_, *aacA4, aadA2*, *ant*(2’’)*-Ia*, *aph*(3’)*-IIb*, *strA*, *strB*, *sul1*	*algD, aprA, lasB, plcH*, *plcN*, *exoT*, *exoU*, *exoY*, *phzM*, *phzS*, *phzH*, *pilB*, *toxA*	-
Pae90-Lar	235	No	-	*bla*_PAO_*, bla*_OXA-35_, *bla*_OXA-488_, *aacA4*, *aph*(3’)*-IIb*, *catB7*, *sul1*	*algD*, *aprA, lasB*, *plcH*, *plcN*, *exoT*, *exoU*, *exoY*, *phzM*, *phzS*, *phzH*, *pilB*, *toxA*	Type I-C
Pae91-Lar	244	No	-	*bla*_PAO_*, bla*_OXA-494_, *aph*(3’)*-IIb*, *catB7*	*algD*, *aprA*, *lasB*, *plcH*, *plcN*, *exoT*, *exoU*, *exoY*, *phzM*, *phzS*, *phzH*, *pilB*, *toxA*	Type I-F
Pae462-Lar	253	No	-	*bla*_PAO_*, bla*_OXA-488_, *aph*(3’*)-IIb*, *catB7*	*algD*, *aprA, lasB*, *plcH*, *plcN*, *exoT*, *exoU*, *exoY*, *phzM*, *phzS*, *phzH*, *pilB*, *toxA*	Type I-F
Pae100-Lar	299	No	-	*bla*_PAO_*, bla*_OXA-494_, *aph*(3’)*-IIb*, *catB7*	*algD*, *aprA*, *lasB*, *plcH*, *plcN*, *exoS*, *exoT*, *exoY*, *phzM*, *phzS*, *phzH*, *pilB*, *toxA*	-
Pae86-Lar	446	No	-	*bla*_PAO_*, bla*_OXA-395_, *aph*(3’)*-IIb*, *catB7*	*algD*, *aprA*, *lasB*, *plcH*, *plcN*, *exoT*, *exoU*, *exoY*, *phzM*, *phzS*, *phzH, pilA*, *pilB*, *toxA*	-
Pae64-Lar	664	No	-	*bla*_PAO_*, bla*_OXA-50_, *aph*(3’)*-IIb*, *catB7*	*algD, aprA, lasB, plcH*, *plcN*, *exoS*, *exoT*, *exoY*, *phzM*, *phzS*, *phzH*, *pilB*, *toxA*	-
Pae475-Lar	2048	No	-	*bla*_PAO_*, bla*_OXA-494_, *aph*(3’)*-IIb*, *catB7*	*algD, aprA, lasB, plcH*, *plcN*, *exoS*, *exoT*, *exoY*, *phzM*, *phzS*, *phzH, pilA*, *pilB*, *toxA*	-

^a^ The absence of a CRISPR/Cas system is indicated by a ‘-’.

**Table 3:** Susceptibility levels of carbapenems and ciprofloxacin, and amino acid substitutions in the GyrA, GyrB, ParC, ParE, MexR, NfxB, MexT and OprD amino acid sequences of 18 *P. aeruginosa* characterized by Illumina sequencing.

Isolate	ST	MBL Content	MIC (μg/mL) ^1^			Amino Acid Changes ^2^							
			Imp	Mem	Cip	GyrA ^3^	GyrB ^4^	ParC	ParE	MexR	NfxB	MexT	OprD ^5^
Pae8397-Lar	111	VIM-2	>16	>16	>4	T83I, V671I, G860S, D893E, A900G, S903A, ^+^S912, ^+^E913	SB1	S87L, F254V, A346Q	T89I, I91T	V126E	E124A	L26V	* 94
Pae9981-Lar	111	VIM-2	>16	>16	>4	T83I, V671I, G860S, D893E, A900G, S903A, ^+^S912, ^+^E913	SB1	S87L, F254V, A346Q	T89I, I91T	V126E	E124A	L26V	Dis_337_
Pae8709-Lar	235	VIM-4	>16	>16	>4	T83I	SB1, E469D	S87L, F254V, A346Q	T89I, I91T, D533E	V126E	E124A	L26V	* 94
Pae9938-Lar	235	VIM-4	>16	>16	>4	T83I	SB1	S87L, F254V, A346Q	T89I, I91T, D533E	V126E	E124A	L26V	Non-SC
Pae8414-Lar	395	VIM-2	>16	>16	>4	T83I, ^+^E909, ^+^S910	SB1	S87L, F254V, A346Q	T89I, I91T, V200M	-	E124A	L26V	* 229
Pae9047-Lar	395	VIM-2	>16	>16	>4	T83I, ^+^E909, ^+^S910	SB1	S87L, F254V, A346Q	T89I, I91T, V200M	-	E124A	L26V	Non-SC
Pae112-Lar	162	No	>16	>16	1	T83I	SB1	F254V, A346Q	T89I, I91T, A473V, D533E	-	E124A	L26V	* 219
Pae69-Lar	235	No	>16	>16	>4	T83I	SB1, P750S	S87L, F254V, A346Q	T89I, I91T, D533E	V126E	E124A	L26V	FR_501_
Pae90-Lar	235	No	>16	>16	>4	T83I	SB1	S87L, F254V, A346Q	T89I, I91T, D533E	V126E	E124A	L26V	* 142
Pae91-Lar	244	No	>16	4	≤0.25	-	SB1	F254V, A346Q	T89I, I91T	-	E124A	L26V	Non-SC
Pae462-Lar	253	No	>16	>16	1	^+^E909, ^+^S910	SB1	F254V, A346Q	T89I, I91T, A473V, D533E	G91E, V126E	R21H, D56G, E124A	L26V	FR_501_
Pae100-Lar	299	No	>16	8	≤0.25	-	SB1	F254V, A346Q, V646L	T89I, I91T, D533E	-	E124A	L26V	Non-SC
Pae86-Lar	446	No	>16	4	2	^+^E909, ^+^S910	SB1	F254V, A346Q, A587T	T89I, I91T, D533E	V126E	E124A	L26V	Non-SC
Pae64-Lar	664	No	2	12	1	-	SB1	F254V, H262Q, A346Q	T89I, I91T, E215Q	* 94	E124A	L26V	Non-SC
Pae475-Lar	2048	No	>16	4	≤0.25	-	SB1	F254V, A346Q	T89I, I91T	-	R82L, E124A	L26V	* 424

^1^ Imp, imipenem; Mem, meropenem; Cip, ciprofloxacin. ^2^ Sequences were compared to those under GenBank accession no. L29417 for GyrA, AB005881 for GyrB, AB003428 for ParC, AB003429 for ParE, U23763 for MexR, X65646 for NfxB, AJ007825 for MexT, and AE004091 for OprD. ^3^ The duplication of the specific amino acids is shown by +. ^4^ SB1 indicates substitution of amino acids G151-S152-A153-V154-P155-T156-A157-R158-S159-G160-R161-R162 to V151-P152-Q153-F154-P155-L156-R157-E158-V159-G160-E161. ^5^ The coding sequences for OprD were analyzed for the presence of stop codons. Non-SC symbolizes the absence of a stop codon in the deduced protein sequences. * shows the stop codon position in the deduced protein sequences. Dis symbolizes disruption of *oprD* locus, while FR shows the presence of a frameshift resulting in a longer OprD protein. Mutations that have been described previously to confer resistance to carbapenems are indicated in bold.
